# Psychosocial Aspects of Female Breast Cancer in the Middle East and North Africa

**DOI:** 10.3390/ijerph17186802

**Published:** 2020-09-18

**Authors:** Haya Salem, Suhad Daher-Nashif

**Affiliations:** 1 Basic Medical Sciences Department, College of Medicine, QU-Health, Qatar University, Doha 2713, Qatar; hm1602810@student.qu.edu.qa; 2 Population Medicine Department, College of Medicine, QU-Health, Qatar University, Doha 2713, Qatar

**Keywords:** breast cancer, women, culture, well-being, quality of life, Arab region

## Abstract

Breast cancer, the most common cancer among women in the Middle East and North Africa (MENA) region, is associated with social and psychological implications deriving from women’s socio-cultural contexts. Examining 74 articles published between 2007 and 2019, this literature/narrative review explores the psychosocial aspects of female breast cancer in the MENA region. It highlights socio-cultural barriers to seeking help and socio-political factors influencing women’s experience with the disease. In 17 of 22 Arab countries, common findings emerge which derive from shared cultural values. Findings indicate that women lack knowledge of breast cancer screening (BCS) and breast cancer self-examination (BSE) benefits/techniques due to a lack of physicians’ recommendations, fear, embarrassment, cultural beliefs, and a lack of formal and informal support systems. Women in rural areas or with low socioeconomic status further lack access to health services. Women with breast cancer, report low self-esteem due to gender dynamics and a tendency towards fatalism. Collaboration between mass media, health and education systems, and leading social-religious figures plays a major role in overcoming psychological and cultural barriers, including beliefs surrounding pain, fear, embarrassment, and modesty, particularly for women of lower socioeconomic status and women living in crises and conflict zones.

## 1. Introduction

Breast cancer is the leading type of cancer for females across the Arab (MENA) region. Adib et al. [1] noted that 30.1% of female cancer cases are breast cancer, while El-Attar [2] found that breast cancer comprises between 16.2% and 38.4% of all types of cancer. In second place on the prevalence scale is non-Hodgkin lymphoma (6.8%), followed by rectal (6.1%) and thyroid (5.7%) cancers. The least prevalent are buccal, gallbladder, and bladder cancers (0.7%). Meanwhile, earlier studies found that colorectal cancer is the second most prevalent after breast cancer among women in some Arab countries [3,4]. While many studies in the MENA region focused on prevalence, epidemiology, and etiology of female breast cancer, several studies highlighted the psychosocial experiences of the women who had been diagnosed. This study aims to review these studies and identify the main socio-cultural and demographic factors affecting Arab women’s psychosocial experiences through their journey with breast cancer.

In their study of the epidemiology and management of breast cancer in Arab countries, El Saghir et al. [5] reported that people in the region are reluctant to speak openly about cancer and that it is highly stigmatized, to the extent that many refrain from mentioning it by name. Hence there is a need to better understand the cultural and religious landscape in which breast cancer patients undergo their treatment, the specificities of which may influence their subjective experiences. Recent studies have shown a positive impact of early examination, which leads to early diagnosis and breast-conserving surgery [5]. Bener et al. [6] found that health workers rarely recommend examinations, resulting in women having inadequate knowledge about breast cancer screening [7,8]. Despite breast cancer being one of the most common cancers among women, the uptake of breast cancer screening (BCS) and breast cancer self-examination (BSE) is still relatively low. Donnelly et al.’s [9] multicenter cross-sectional quantitative survey conducted amongst Arab women in Qatar reported that in addition to the low levels of awareness and participation in BCS, only one-quarter of the participants reported that their doctors discussed and recommended BCS. Women who did not undergo BCS cited as reasons the lack of physicians’ recommendation, fear, and embarrassment [7,9]. Donnelly et al. [9] attribute late diagnosis of the disease to the low awareness of the importance of BCS. They link this low awareness to a variety of factors, mainly age, education, and the absence of a doctor’s recommendation.

A diagnosis of breast cancer can be disruptive to an individual’s physical, psychological, interpersonal, and financial states [10]. Breast cancer and its treatment are associated with a broad range of symptoms that impact physical, social [11], mental (especially when self-esteem is influenced by changes in the body), and cognitive functioning. This upheaval can have a profound impact on women’s well-being and quality of life. Within clinical domains, quality of life has become an important health outcome measure, used to indicate which facets of an individual’s life are most affected by a disease and its treatment [12]. Wisloff et al. [13] demonstrate a link between compromised quality of life and clinical outcomes, including survival. Sociodemographic factors emerge as an important theme in the quality of life literature [14]. Past studies found that factors such as a patient’s educational level, age, amount of spousal and familial support, employment and financial status, and stage of disease progression can predict the quality of life of patients with breast cancer [10,15,16]. In this paper, we present the main psychosocial aspects of being a woman with breast cancer in the MENA region.

## 2. Materials and Methods

This study is a narrative literature review, in which the researchers reviewed existing studies on the psychosocial aspects of female breast cancer in the MENA region published between 2007 and 2019. We chose a literature review because we aimed to document the main findings by thematically analyzing and integrating the knowledge gained over a whole decade by several researchers in all Arab countries. In this type of review, researchers can summarize shared themes across existing studies, bringing together their conclusions into a holistic interpretation contributed by the reviewers In doing so, we can also summarize similarities and differences, meaning that while we consider the socio-cultural similarities shared by the Arab countries, we can also explore the uniqueness of a specific country that results from its local circumstances. Literature review studies’ results are narrative and qualitative rather than quantitative, and they enable the researcher to acknowledge, reflect, and assist the reviewed data. Using a literature review allows us to analyze the psychological and socio-cultural barriers, needs, and challenges facing Arab women with breast cancer.

**Figure 1:**
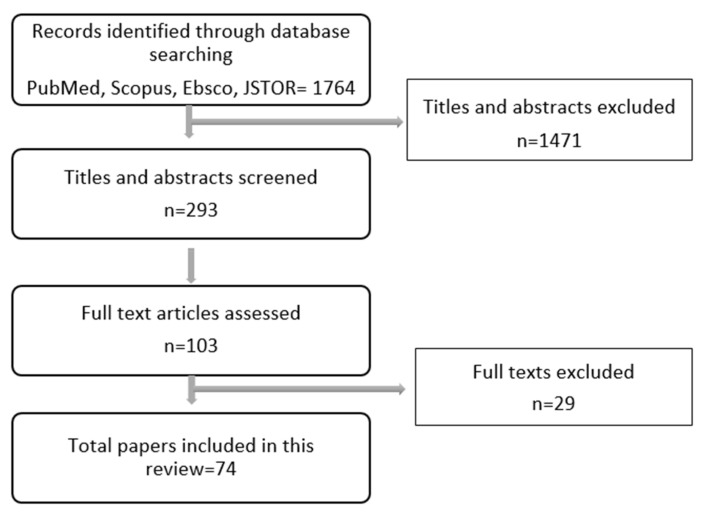
Flowchart of research process.

We began by searching academic databases including PubMed, Scopus, Ebsco, and JSTOR. We used the keywords “society”, “culture”, “breast cancer”, “women”, “screening”, “quality of life”, “attitudes”, “stigma”, “women”, “Arab”, “Muslim”, “family”, “coping”, “mental health”, and “religion”. The study included only English language articles and covered both qualitative and quantitative studies. Chapters in books, research reports, Arabic articles, and studies and information published as abstract only were excluded. Both authors, with the assistance of two students, reviewed the titles and abstracts gathered. We found 293 candidate articles, which were further reviewed and reduced to 74 articles (Figure 1; Table S1).

We included only research papers where the main topic was psychosocial experiences and psychosocial aspects of female breast cancer in the MENA. In order to analyze and categorize the content of the articles included, we used conventional content analysis whereby identifying the main concepts and categories is derived directly from the text data [17]. In this type of analysis, researchers determine the presence of certain themes or concepts within the given qualitative data (i.e., text), that repeat themselves across the data. By using content analysis, researchers can identify the presence of certain themes and concepts, as well as identify meanings and relationships between the identified themes and concepts. For credibility assurance, the authors debriefed and discussed the methods and results with experts in the topic of this review from different health sciences fields. The team of experts included two public health researchers, a psychologist, a psychiatrist, a social sociologist, and a medical anthropologist. Furthermore, the researchers evaluated the quality of their review by using the SANRA scale. SANRA Scale is a validated tool developed to evaluate the quality of narrative and literature reviews [18]. The scale includes six items: Justification of the article’s importance, Statement of concrete aims of the review, Description of the literature research, Referencing, Scientific reasoning, and Appropriate presentation of data. Each item includes three levels, zero, one, and two, where two indicates that the review meets the criteria for quality. We evaluated the manuscript individually and then met to discuss gaps and ways to improve the manuscript in order to meet SANRA’s criteria.

## 3. Results

In reviewing the 74 papers on our final list, we identified five main themes that highlight the psychosocial aspects of female breast cancer in the MENA region. These themes are 3.1. Who cares? 3.2. Sociodemographic and cultural determinants associated with breast cancer, 3.3. Awareness and sources of knowledge, 3.4. Quality of life of women with breast cancer, and 3.5. Family support and relationships of women who have breast cancer.

### 3.1. Who Cares? States, Themes, Types, and Subjects of the Studies Included

**Figure 2:**
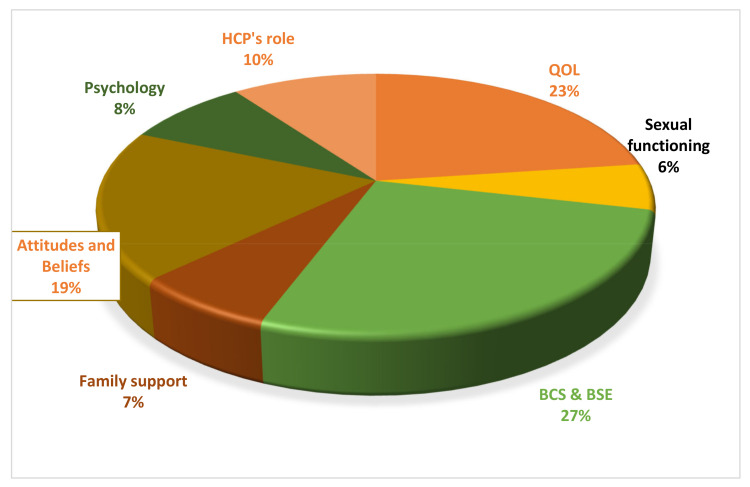
Topics of discussion in studies on breast cancer.

BSC/BSE was the most frequent topic, discussed in 27% of studies. This was followed by the topic of quality of life of women with breast cancer (23%). The least discussed topic was sexual functioning (6%). Other prevalent themes included attitudes and beliefs regarding breast cancer, its examination and its treatment (19%); health care providers’ roles (10%); and family support and mental health issues (8%) (Figure 2).

**Figure 3:**
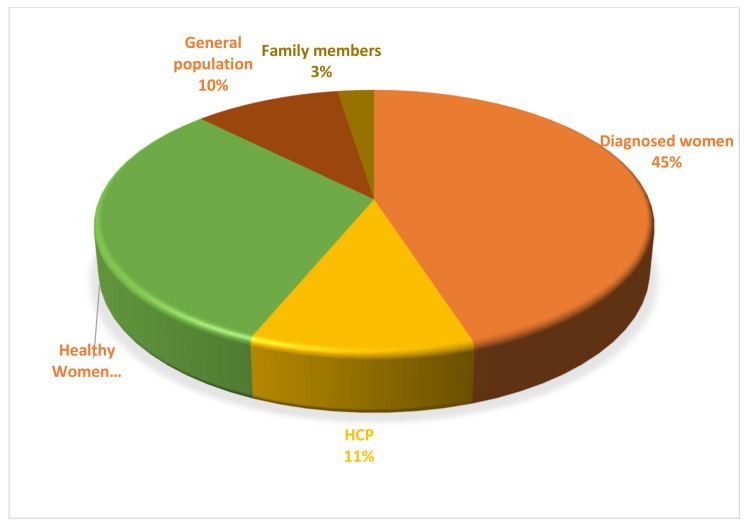
Categories of research participants in studies used.

Some countries conducted a higher number of studies than other countries. Egypt, Lebanon, Palestine, Qatar, and Saudi Arabia were amongst those who published the highest number of studies on the addressed issues. Libya, Syria, Yemen, and Sudan were amongst those who published the lowest numbers of studies. North Africa’s countries such as Tunisia and Morocco were in the middle. North African countries (Tunisia, Morocco, Algeria, and Mauritania) are considered francophone countries, and researchers in these countries publish in French more than in English. It is therefore likely that these countries have additional studies published in French, but these were not included in our review due to the exclusion criteria. Among the Middle Eastern countries, Syria, Libya, and Iraq had the lowest number of studies. This can be explained by the political crises and conflicts these countries have been experiencing since 2011, which have made conducting research more difficult. The most frequent research participants were diagnosed women, included in 45% of studies, healthy women (31%), health care workers (11%), the general population (10%), and family members (3%) (Figure 3).

Most of the studies were quantitative (70%), 20% were qualitative, and 10% were review studies. We argue that the number of the studies, the subjects, and the topics indicate who cares about which aspect of psychosocial experiences of female breast cancer.

### 3.2. Sociodemographic and Cultural Determinants Associated with Breast Cancer

The studies reviewed in this paper indicate that an alarming number of women received their breast cancer diagnoses later in the course of their disease (Stage III and IV). Socio-cultural and political factors, family values, and religious beliefs were consistently mentioned by the studies as the main factors contributing to women being late in seeking help and thus receiving a late diagnosis.

Economic status and political situation were reported as the most influential factors with regard to access to health care. The reviewed studies indicated that a lack of health insurance, low economic status/income, and distance from health care facilities (being unable to pay for transportation or accommodation near hospitals) seemed to be the most prominent causes of late diagnosis [1,19,20,21]. Conflicts and political crises were an additional reason for lack of access to healthcare systems. For example, Palestinian women living in the West Bank faced hardships passing military checkpoints. As a result, they considered the journey to a hospital for screening worthless and postponed seeking diagnosis until the late stages of the disease [22,23].

Religious beliefs were also found to have an impact on women’s experiences. When women were questioned about their assumptions with respect to the causes of breast cancer, many linked it to religious causes, such as a test or punishment from God for previously committed sins [24,25,26]. In some contexts, women mentioned unique beliefs regarding the causes of breast cancer, including the increased use of hormonal birth control pills and not breastfeeding. Women believed that Islam promotes and encourages women to breastfeed and utilize natural birth controls for several reasons. One is to protect against breast cancer [25,26]. Furthermore, cultural norms and religious beliefs constituted barriers to early diagnosis and treatment [27,28]

Religious practices, such as becoming more devout and praying much more often, as a means of coping with their illness were reported by women, as well as references to *tawakkul* (trust in God) [29,30,31,32]. In addition, screening and early diagnosis were perceived to be worthless and futile since God is the only protector and healer, even though, opposingly, treatment was claimed to be necessary since “our bodies are lent to us by God” and must be taken care of [25,26,33]. A Moroccan study that interviewed nurses and doctors showed that 60% of nurses believed that breast cancer can be cured by adhering to prayer without any kind of therapy [34]. Moreover, screening was considered to be a breach of a woman’s Islamic modesty [35,36].

Some cultural values in the MENA region necessitated having a female doctor for clinical breast examinations (CBE) and mammograms, which resulted in a delay in diagnosis of approximately 8 months among Libyan, Palestinian, and Egyptian women [25,37,38]. Furthermore, culturally, the term “cancer” has been shown to be associated with either death or baldness due to chemotherapy [29,31]. This seemed to be a more common conception among Arab women, leading them to avoid mentioning it to their families. For example, Palestinian women living inside Israel reported that they felt more comfortable communicating with Jewish women during their chemotherapy sessions than with fellow Palestinians who also suffered from breast cancer [39]. Their comfort speaking about their diagnosis with strangers resulted from the social stigma and the way that cancer is perceived and framed by their close community. Cancer was also linked to being attacked by someone’s “Evil Eye”, and was believed to be able to be resolved on its own or through the use of home remedies, such as rubbing the lump with olive oil [24,29,32,40,41,42,43].

### 3.3. Awareness and Sources of Knowledge

Knowledge about breast cancer risk factors and clinical features is a vital indicator of conducting regular BSE, seeking CBE, and pursuing treatment. Women, mostly working mothers, who had knowledge about BSE practice lacked general information about the frequency and best time of their menstrual cycle to perform BSE [44,45,46,47]. Knowledge of some indicators of breast cancer risk factors, such as the absence of non-lump breast signs, nipple retraction or changes in breast size and/or shape, other health symptoms like weight loss or fatigue, and denial of and fear of finding a lump, made women less likely to perform BSE or undergo a CBE or mammogram [20,27,34,37,42,48,49,50]. Furthermore, women who successfully underwent a BSE, CBE, or mammogram once did not repeat the screening tests regularly since they believed that the initial negative results accompanied by no breast changes made a second screening unnecessary [36,51,52]. For example, it was found that in the Palestinian Authority, more than 60% of women above the age of 50 had never undergone a mammography and did not know about the need for regular screening tests, while 72% had never had a CBE [22]. Women who underwent CBE also performed BSE regularly, which was also a common trend among Qatari women [26]. It was also noted that women who performed screenings were motivated to do so because they knew someone (a family member or friend) who had suffered from breast cancer [22,26,32,36,45]. The gap between initial occurrence of symptoms and diagnosis among women of different Arab nationalities living in the UAE ranged from 3 months to 3 years, and this was largely explained by the inappropriate information that women had about the presentation of breast cancer [42]. A huge gap in the knowledge of the risk factors of breast cancer was also noticed among women in northern Saudi Arabia, which may have resulted in delayed diagnosis, as in the UAE [53]. On the other hand, 15.5% of Libyan women reported that they had been falsely reassured by their physicians that their breast lump was benign [37].

The impact of level of education on breast cancer awareness is very debatable. Despite the expectation or hypothesis that women who attended university or who had higher levels of education would have had more knowledge about breast cancer diagnosis, screening, and risk factors, the actual correlation varied between countries. For example, female students attending the University of Assuit and Ain Shams in Egypt and Taibah University in Saudi Arabia were found to have poor knowledge about breast cancer risk factors and clinical features [48,54,55]. Even among those students who had a good level of knowledge about breast cancer and were aware of the existence of BSE, the majority did not recognize the need to perform screenings regularly because of their young age or lack of knowledge on how to perform it. This lack related to a low interest in learning about the topic. Similar results were found in a study of Sudanese medical students who were trainees in the OB/GYN department at Omdurman Maternity Hospital [56]. Likewise, among Omani women who had completed postgraduate studies, almost half had poor knowledge about breast cancer. Similar results were also observed among Jordanian female students of different majors and departments [57].

In addition to the deficiency in information, women reported that their primary source of information was not from health care providers but rather from the media, including the internet, social media, and television [9,26,32,44,45,47,58,59,60,61,62]. More than half of the subjects in the papers who underwent BSE, CBE, and/or mammograms were advised by the doctors whom they trusted the most. In Iraq, although both students and teaching staff at the Technical Institute of Shatra showed a high percentage of awareness, 73% and 88%, respectively, only 25.4% and 24.4%, respectively, performed BSE [63]. The participants of this study also reported that their principal sources of information (from the most to least popular) were the internet and television (47%), health care providers (27%), and family (26%) [63]. In Palestine, only 15% of doctors recommended breast exams to their patients; 48% of them remarked that they did not know if radiotherapy was available to women in Gaza. Similarly, only 19% of female practitioners in Saudi Arabia ordered a mammogram for women over 40 years of age despite their high level of knowledge about breast cancer risk factors [64]. Therefore, the lack of physicians’ recommendations for screening was highlighted in most of the countries. Researchers attributed this lack to the embarrassment and shame that derive from gender and body values within the discussed societies [26,37].

Researchers found that the patient’s level of education influenced the level of knowledge of risk factors as well as the way women coped with the diagnosis. Women who had a university degree or higher, especially those who were working, tended to experience less severe episodes of depression and anxiety and had better physical functioning [1,65,66,67,68,69]. In other words, knowledge and education were found to be helpful in improving women’s quality of life (QOL). Hence, we can conclude that increasing awareness and knowledge around breast cancer can be an alternative to physician recommendation.

### 3.4. Quality of Life of Women with Breast Cancer

According to the WHO, quality of life (QOL) is defined as an individual’s perception of their position in life within the context of the culture and value systems in which they live and in relation to their goals, expectations, standards, and concerns [70]. When Arab women were asked about their definition of quality of life, they generally described their ability to fulfill their roles as mothers and wives [24,28,29,33,71]. Lower income and higher financial stress were associated with a poor quality of life in addition to higher pain severity [71]. In Yemen, younger women had worse scores on the quality of life scales [72]. This could be due to the socio-political crisis in which they live, which is described by many human rights organizations as the worst crisis of the century. Adverse effects related to treatment interfered with general activity and social functioning and hindered women from performing their day-to-day chores as mothers and wives [24,33,71,73,74]. Women described physical symptoms, including fever, nausea, fatigue, vomiting, dyspnea, poor appetite, and arm pain, which greatly impacted them psychologically, leading to depression, constant anxiety, sleep disturbances, and social difficulties. The reverse effect was also evident [67,75,76,77,78]. Studies revealed that the extent of pain was influenced by the time of diagnosis as well as the mode of treatment. Women who underwent radiation and/or immunotherapy scored best on a spiritual well-being scale [79,80]. Studies in Egypt and Jordan showed that physical and psychosocial symptoms were statistically significantly better in those who underwent breast-conserving surgery rather than modified radical mastectomy [66,81]. In Jordan, lumpectomy surgeries had better outcomes compared with mastectomies [66]. The studies indicated that the progression of women’s psychiatric symptoms was significantly associated with advanced stages of the disease, such as metastasis, breast cancer relapse, and multiple tumors, and with continuous post-treatment pain [67,71,77,80,82]. Hence, the stage of the disease was a direct factor in shaping their perception of quality of life. Some of the issues commonly reported by women as consequences of therapy, that their physicians failed to mention, were loss of what represented their femininity, such as breast resection, hair loss, breast pain, lack of desire to engage in sexual activities, and vaginal dryness [24,29,66,74,83,84]. In Morocco, for example, 84% of women with breast cancer who continued sexual activity described it as being increasingly uncomfortable/unenjoyable, painful, and undesirable [77,78,83]. This was substantiated by research done in Bahrain in which women were interviewed about their spouses’ reactions to their illness. The women expressed that they felt rejected and weak [85]. Another study in Bahrain showed that husbands sometimes even interfered with the type of treatment their wives were to undergo by insisting on less aggressive forms of treatment or none at all [24]. This indicates that women’s quality of life is partially shaped by gender hierarchies within their society. Some women tried to tackle the decline in their quality of life caused by breast cancer by resorting to alternative medicine (such as special foods, herbs, supplements, spiritual activities) in parallel with, and sometimes instead of, their treatment plans due to a fear of the toxicity caused by chemotherapy. A high percentage of those women did so without consulting their doctors [43,86]. 

### 3.5. Family Support and Social Relationships of Women Who Have Breast Cancer

The studies that were reviewed showed the importance of the role of the family in both diagnosis and treatment stages. For example, divorced and widowed Saudi women scored lower on social well-being than their married counterparts [79]. Patients were affected by the presence or absence of familial support as well as their families’ reactions to the illness. An example of the importance of familial support was illustrated in a study performed by Adib et al. [1], which showed that Iraqi migrants living in Lebanon suffered from depression and anxiety more than Lebanese women. This was mainly due to the lack of nuclear family, extended family, and community support, in addition to other factors such as economic status. Furthermore, family structures often change when women are unable to perform their roles as wives and/or mothers [31,40].

While the majority of women shared the news of their diagnosis with their closest family members, other women felt obliged to hide the news from their family members for various reasons. For example, some hid it from their husbands due to the fear of divorce or being forced to accept their husbands marrying a second wife [33,84]. This behavior resulted from the impact of the breast cancer diagnosis and its implications for women’s femininity, marriage, intimate relationships, and body image [33,40]. Women hid their diagnosis from their friends due to their fear of becoming a burden and of the social stigma associated with “that” disease. This made women hesitant about mentioning their hospital visits to avoid questions from people who knew them [33,39]. Hiding their diagnosis from family and friends led women not to receive the appropriate treatment in time because they were unable to hide the side effects of ongoing treatment [39].

Some studies mentioned that women hide their diagnosis from their children to protect them from worry and grief. For example, mothers in Saudi Arabia who revealed their diagnosis to their children, despite experiencing a stronger mother–child bond, admitted that the news negatively affected their children’s academic performance, which in turn increased the burden on the women [87]. Hiding a diagnosis from children is a protective act resulting from mothers’ fear of negatively impacting their children’s emotional and academic performance. This is a mothering value, protecting the child despite being in need of support oneself.

## 4. Discussion

This review found that several socio-cultural and political factors affect Arab women’s quality of life when they are diagnosed with and treated for breast cancer. One of the interesting findings was that few studies addressed sexual functioning, family support, and mental health. These are essential needs that can be reflected directly in women’s QOL in the process of healing and facing cancer. We argue that researchers and health care workers should give these factors as much attention as they do the physical symptoms. We assume that the absence of studies on sexual functioning indicates that the sexuality, sexual health, and sexual functioning of women and their ability to express their attitudes and feelings toward it are still considered inappropriate or taboo [88]. Similarly, the social stigma surrounding mental health constitutes a barrier to seeking help for the patient, and a barrier to researchers in asking patients about the issue [89]. In Arab societies, family members tend to hide illness and disease from both their close community and strangers because the cultural value is to keep family issues in the home [89]. This can be explained by the fact that illness and disease are considered weaknesses that society must not see, because if they are seen, the family will be perceived as weak and vulnerable by society.

The political events since 2011, beginning with the “Arab Spring”, led to continuous crises in several countries, including Syria, Libya, Yemen, and Iraq. We assume that these events have affected women’s life in general and have influenced the research agenda within these countries, which reflected by the scarcity of studies on women and breast cancer. In general, in times of conflict women become more vulnerable and women’s issues are pushed to the bottom of national priorities [90]. Sidel and Levy [91] argue that armed conflicts may be associated with poor health and poor access to quality medical care, especially for women, children, and the elderly, that is, the vulnerable and those most affected by wars and conflicts. Political conflicts have a direct impact on women’s QOL, healing processes, and the general ability to cope with breast cancer. Hence, these women are essentially facing three battles: political, medical, and social. Wars reproduce patriarchy within and between genders and thus require a focus on those institutions that are crucially responsible for the production of masculine identity [90].

Almost half of the studies we reviewed focused their research on women who had been diagnosed with breast cancer, while one-third studied the experiences and attitudes of healthy women. This reflected the patient-centered approach in the studies, where women’s voices were the main voices. Despite this, most of the studies were quantitative. While quantitative methodologies tend to analyze phenomena in terms of prevalence and frequencies, qualitative methodologies aim to determine the meaning of a phenomenon and develop concepts that help in our understanding of the phenomenon through those who are involved in it, that is, the human participants. We attest that there is a need for more qualitative or mixed-methods studies in order to better understand women’s experiences and needs. This will help to better implement the patient-centered approach in healthcare systems, which highlights patients’ perspectives as a basic principle in health care [92].

Socio-cultural factors, such as religion and cultural values, were found to play a major role in BSE and BCS behaviors. The term “cancer” has a negative connotation because it is associated with death and end of life. In her work *Illness as Metaphor* (1978), Susan Sontag clarifies how fatalistic social perceptions and the framing of cancer, reflected in the language and metaphors used to refer to the disease, negatively influence women’s perceptions, experiences, and healing processes [93]. The Health Belief Model [94] considers cultural values and religion as significant variables affecting perceptions of illness and health, with a major impact on managing health and sickness in certain societies. In Arab-Muslim societies, that is all MENA states, religion plays a role on three levels: the holy text (Quran and Hadith), the belief system that derives from the text, and the social-religious practices that derive from both [95]. In several contexts, there may be gaps between the holy text, the belief system, and the religious practice. In our review, the gap was between the perception of illness as a punishment or test that only God can heal, and the belief that our bodies are a gift from God and we have to take responsibility and care for them. This contradiction is reflected in women’s screening behavior whereby they say that God gave us our bodies and it is our duty to care for them, yet they also engage in late screening behaviors resulting from fear, embarrassment, and rejection of the idea of being sick. Another example of a gap between the Quranic text and social beliefs is that envy is mentioned in the Quran, the holy book for Muslims, as something people should protect themselves from, but it is not referred to or based on the concept of the “Evil Eye.” Arab-Muslim societies use the Evil Eye to refer to envy of others and every bad event they cannot explain. Abu-Rabia argues that the belief in the Evil Eye is embedded in the folklore of *fallahin* (peasant) societies throughout the Middle East [96]. We argue that this belief in the Evil Eye influences perceptions of disease and illness, and affects the way women with breast cancer, and indeed other ill Arab-Muslim people, manage their diseases. Religious and social leaders, as well as awareness campaigns in all platforms and forms, play a crucial role in re-structuring belief systems and encouraging women to seek early diagnosis and care for their health. The primary factor to consider in preparing these awareness campaigns is the access of women. Access, in this context, refers to language and location.

Studies that considered the role of breast cancer awareness campaigns and investigated their impact on women reflect that these campaigns were ineffective in countries where they occurred abundantly. Women in our review, such as women living in Qatar and Saudi Arabia, recommended using the Arabic language to distribute information. Another common recommendation was to make the campaigns more culturally sensitive by locating informational booths in female-restricted areas instead of public malls, so that women would feel less exposed while visiting the booths [21,38,97]. In addition, in Lebanon it was found that public information campaigns were occurring at a much lower rate in rural areas compared with in the cities [1]. Women in rural areas were found to use more alternative and herbal medicine due to their distance from main health care centers. Few studies mentioned alternative medicine, especially herbal remedies, as a tool women use during their trials to promote healing in parallel to the clinical treatment. Azaizeh et al. [98] found that the eastern region of the Mediterranean has been distinguished from other regions through a rich inventory of complementary alternative medicine, in particular herbal medicine.

Our review found that being a working and educated mother does not necessarily indicate better self-care or better awareness of BSE; in fact, we found that knowing someone, such as a family member or friend, who was diagnosed with breast cancer had a stronger impact on self-health management, resulting from fear that prompted women to perform self- or clinical screening. Similar results were found in non-Arab countries, such as in Serbia [99]. We assume that many Eastern and Western societies may express fear as an emotional reaction associated with the diagnosis of a close friend or family member. The difference between societies is in the way their culture shapes an individual’s reaction to it.

One of the cultural factors found to create delay in seeking diagnosis was a woman’s preference in having a female doctor perform the clinical breast examination. The best way to counter this factor is to seek out female doctors. The healthcare system should also construct a culturally tailored system by providing more female doctors. In addition to a lack of female doctors available, our review found that the lack of a physician’s recommendation for breast examination was significant. Indeed, this is one of the major reasons women were unaware of the importance of BSE and BCE. Some researchers attributed this lack of physician recommendation to the gender dynamics and cultural values of Arab societies, which can result in embarrassment and avoidance by doctors who would be recommending BSE or BCE to their patients. Lack of doctors’ support and women’s preference not to discuss health concerns with family members led them to rely on the media, social media, and the internet as their main resources of knowledge. Although some cultural values created barriers to seeking early diagnosis, other cultural values promoted the healing processes. For example, the Arab cultural structure is characterized by collectivism and patriarchy, which was found to play a positive role in the way women were able to deal with treatment. Women reported relying on support from their extended families that lived nearby when they were in need. Extended family members filled the woman’s roles in mothering, housekeeping, and managing the daily lives of her nuclear family. Studies from other regions in the world found the same results in terms of extended family support [100,101]. Furthermore, we found that migrant women fleeing war, such as Iraqis, Syrians, and Yemenis who lived in other Arab countries far from their extended families, lacked the necessary support and this was reflected in their poorer quality of life.

## 5. Conclusions

Socioeconomic and socio-cultural factors are important in shaping the quality of life of Arab women with breast cancer. These factors are combined with clinical factors, such as the stage of disease and treatment modality. While all women with breast cancer generally share these experiences and impacts, the features of the society that Arab women inhabit make the development of these experiences unique to them. Gender hierarchies and patriarchy, family values and tribal mentality, cultural practices that contradict the holy religious texts, and cultural perceptions of cancer and women’s bodies may make the experience of being diagnosed with and treated for breast cancer harder for women living in the MENA. A limitation of this study is that it may not have included all relevant studies because the search was limited to four databases. Indeed, literature reviews cannot include every study on the topic, and this is one limitation of this type of review. We also think that including the studies published in Arabic and French, especially on North Africa’s countries, could enrich this review. Further qualitative studies are recommended to explore women’s actual lived experiences, especially during the current COVID-19 outbreak. Such studies may shed light on how women’s psychosocial experiences are affected during an infectious disease outbreak, a research gap that should be explored during this painful but historical era of COVID-19.

## Supplementary Materials

The following are available online at https://www.mdpi.com/1660-4601/17/18/6802/s1. Table S1: The included studies.
