# Supplementary material for: Psychosocial Aspects of Female Breast Cancer in the Middle East and North Africa
Source: Int J Environ Res Public Health. 2020 Sep 18;17(18):6802. doi: 10.3390/ijerph17186802 (PMC7559584; doi:10.3390/ijerph17186802)
Supplement: Supplementary file 1 [file ijerph-17-06802-s001.pdf]

Supplementary 1. The included studies

Title	Author/s	Year	Journal	Methods	Subjects	Study's location (country)	Main findings
Knowledge, Attitudes, and Practices Surrounding Breast Cancer and Screening in Female Teachers of Buraidah, Saudi Arabia	Dandash, K.F.; Al-Mohaimeed, A.	2007	Int. Journal of Health Science	Cross sectional study	376 female teachers	Kingdom of Saudi Arabia (KSA)	More than half of the women showed a limited knowledge level. Among participants, the most frequently reported risk factors were non-breast feeding and the use of female sex hormones. The printed media was the most common source of knowledge. Logistic regression analysis revealed that high income was the most significant predictor of better knowledge level. Knowing a non-relative case with breast cancer and having a high knowledge level were identified as the significant predictors for practicing BSE.
Between traditional and modern perceptions of breast cancer and cervical cancer screenings: a qualitative study of Arab women in Israel.	Azaiza, F.; Cohen, M.	2007	Journal of Psychology and Oncology	Focus Group interviews	51 Palestinian women from 5 different Arab villages in Israel. .	Israel	All women knew about breast cancer and screening for early detection for breast cancer but not for cervical cancer. Sources of information were mainly: lectures, electronic media and personal acquaintance and conversations with friends. Knowledge of breast cancer was a mixture of traditional concepts and biomedical views. In the Bedouin group, traditional thoughts like the evil eye were also expressed together with the modern views. There was consensus amongst these women that breastfeeding, giving birth and low-fat rich vegetables nutrition were protective factors against breast cancer. General thought was that cancer is a matter of fate and test that is determined by God. Major barriers to screening were accessibility, language and unfriendly attitudes from the specialists and personal responsibility. Other major factors affecting screening are exposure of body parts when doing the tests, fear from and that women want their husbands to love and accept them and their bodies.

Supplementary 1. The included studies

Breast cancer knowledge, risk factors and screening among adult Saudi women in a primary health care setting-	Amin, T.T.; Mulhim, A.R.S. Al; Meqihwi, A. Al	2009	Asian Pacific Journal of Cancer prevention	Cross sectional descriptive	1,315 Saudi Adult females	KSA	Overall level of knowledge regarding risk factors and appropriate screening was low and dependent upon educational and occupational status. Early screening is underutilized among participants due to several perceived barriers. Clinical breast examinations were employed by less than 5% and mammography by only 3% of cases. A positive family history was found in 18% of cases among first- and second-degree relatives, and 2 % had a prior history of benign breast lesions.
Research in action: Mammography utilization following breast cancer awareness campaigns in Lebanon 2002-05.	Adib, S.M.; Sabbah, M.A.; Hlais, S.; Hanna, P.	2009	EMHJ - Eastern Mediterranean an Health Journal,	Four consecutive annual surveys	1200 Lebanese adult women living in 4 rural areas	Lebanon's rural cities: Akkar and Batroun in north Lebanon, Chouf in the central Mount Lebanon area, Sour (Tyre) in south Lebanon and Zahleh in the eastern inner Bekaa valley.	The utilization of mammography in the previous 12 months was low and increased only slightly over 4 years (from 11% to 18%). In the 2005 campaign, it was twice as high (25%) in greater Beirut than in mostly rural areas, and among women aged 40–59 years (about 21%) compared with younger (12%) or older (11%) women. In each wave, repeat mammograms were less common than first time screening.
Health – related quality of life of Kuwaiti women with breast cancer: a comparative study using the EORTC Quality of Life Questionnaire	Alawadi, S.A.; Ohaeri, J.U.	2009	BMC Cancer	Comparative study using EORTC QoL questionnaire	348 Kuwaiti women with BC	Kuwait	348 participant's women aged 20-81 years; 58.7% had stage III and IV disease. Most (47.8%) met the >66% criterion for good functioning on the BR-23 functional scales. Despite institutional supports, Kuwaiti women had clinically significant poorer global QOL and functional scale scores, more intense symptom experience, in comparison with the international data. Younger women had poorer HRQOL scores. Social functioning accounted for the highest proportion of variance for GQOL.
Factors associated with low screening for breast cancer in the Palestinian Authority: relations	Azaiza, F.; Cohen, M.; Awad,	2010	Cancer		397 Palestinian women	Palestine	Greater than 70% of the women had never undergone mammography or clinical breast examination (CBE), whereas 62% performed self-breast examination (SBE). Women were more likely to undergo mammography if they were less religious and if they

of availability, environmental barriers, and cancer-related fatalism.	M.; Daoud, F.						expressed lower personal barriers (OR, 0.59; 95% CI, 0.29-0.76) and lower fatalism (OR, 0.39; 95% CI, 0.28-0.63). A higher likelihood for CBE was related to being Christian (OR, 2.91; 95% CI, 1.49-5.73) and being less religious (OR, 0.32; 95% CI, 0.13-0.78), to perceived higher effectiveness of CBE (OR, 1.46; 95% CI, 1.20-1.79), and to perceived lower cancer fatalism (OR, 0.35; 95% CI, 0.28-0.60). Women were more likely to perform SBE if they were more educated, resided in cities, were Christian, were less religious, had a first-degree relative with breast cancer, perceived higher effectiveness and benefits of SBE, and perceived lower barriers and fatalism.
Breast Self-Examination: Knowledge and Practice among Nurses in United Arab Emirates	Sreedharan, J.; Muttappillymyalil, J.; Venkotramana, M.	2010	Asian Pacific Journal of Cancer Prevention	Self-administered closed ended questionnaire	154 female nurses	UAE, Ajman	96.1% of the participants were aware of the ideal age to start BSE, while 87.7% respondents knew that women with regular menstruation should perform BSE monthly on a particular day, preferably on the fifth or seventh day after menstruation. With regard to BSE technique, 68.8% knew that both inspection and palpation were the ideal methods to detect any change in the breast. A high proportion, 84.4% of the respondents, reported performing BSE. Among those married, 87.0% and among single 78.3% were practicing BSE.
Awareness and Knowledge of Breast Cancer Among University Students in Al Madina Al Munawara Region.	Habib, F.; Salman, S.; Safwat, M.; Shalaby, S.	2010	Middle East Journal of Cancer	Self-structured questioannaire	301 female students	KSA, Al Taibah University	Their knowledge about the incidence of the disease was poor; only 34% replied correctly. A total of 148 (59.9%) respondents mentioned swelling in the skin/axilla while 123 (49.7%) suggested skin changes as early warning signs of breast cancer. None of the participants expressed knowledge about all established risk factors of the disease. One hundred fifty-nine (64.4%) did not know the proper way to perform a breast self-examination and 104 (42.2%) had never performed this test. Additionally, 128 (51.8%) knew that mammography was a screening tool for breast cancer. Sources of information about the disease were:

Supplementary 1. The included studies

							television and radio (139, 56.2%), printed material in journals and newspapers (86, 34.8%) and family physicians (13, 15.2%).
Knowledge of risk factors, beliefs, and practices of female healthcare professionals towards breast cancer, Morocco.	Ghanem, S.; Glaoui, M.; Elkhoyaali, S.; Mesmoudi, M.; Boutayeb, S.; Errihani, H.	2011	Pan African Medical Journal	Cross sectional study+ questionnaire	136 Cohort of female healthcare professionals in Morocco.	Morocco	Female doctors were the only professional group that had satisfactory knowledge of risk factors while the nurses had an unsatisfactory knowledge with a mean score of 43%. A half of participants believed that that herbal therapy can cure breast cancer. 75% practice breast self-examination once a month and only 15% have ever had a mammogram.
Knowledge, attitude, and behavior among toward cancer preventive practice	Ravichandran, K.; Al-Hamdan, N.; Mohamed, G.	2011	journal of Family and Community Medicine	Cross sectional study from 20 PHCCs	618 males and 719 females Saudis	KSA	Among the female respondents 23.1% reported that they practiced breast self-examination (BSE); 14.2 and 8.1%, respectively, had clinical breast examination (CBE) and mammography. However, 10.0 and 16.1% of the females, aged 40 years and older, reported having had mammograms and CBE, respectively. The BSE performers were more educated, knew someone with cancer, and had heard of the cancer warning signal. Both educational level and 'heard of cancer warning signal' were significantly related to CBE. Cancer information was received from television / radio by 65.1% and from the physician by 29.4%. Even though 69.4% believed that cancer could be detected early, a vast majority (95.8%) felt early detection of cancer was extremely desirable and 55.1% said their participation was definite in any screening program. A majority of the respondents (92.6%) insisted on the need for physician recommendation to participate and 78.1% expected

Supplementary 1. The included studies

							that any such program should be conducted in the existing hospitals / clinics.
Barriers and opportunities for early detection of breast cancer in Gaza women.	Shaheen, R.; Slanetz, P.J.; Raza, S.; Rosen, M.P.	2011	The Breast	Cross sectional survey	100 Gaza women living in Gaza + 55 Gaza women living outside of Gaza	Palestine, Gaza	The survey found that over 90% of both groups were willing to undergo a diagnostic mammogram for a breast complaint and 86% of WIG and 85% of WOG believed survival was increased with early detection. However, only 27% of WIG and 50% WOG were willing to undergo screening mammography. Religion and culture were not barriers to mammography for over 94% of WIG and 98% of WOG. Limited resources and lack of access to medical facilities were identified as barriers in up to 55% of WIG compared to 15% of WOG. Misconceptions about breast cancer were reported more frequently by WIG, including beliefs that breast cancer is not very common and that breast cancer can be contagious.
Public and Professional Educational Needs for Downstaging Breast Cancer in Egypt.	Uddin, N.; Fateem, E.; Hablas, A.; Seifeldin, I.A.; Brown, E.; Merajver, S.D.; Soliman, A.S.	2011	Journal of Cancer Education	Qualitative study with questionnaire and interview	6 focus groups. 48 women	Egypt, Gharbiah Province	Almost all urban and rural women reported that women do not see physicians until they are seriously ill or have advanced cancer. They reported that oncologists or gynecologists were important to be seen first if a woman suspected breast cancer and primary care physician are not the primary line of cancer diagnosis. Other deterring factors besides distrust in primary care physicians included attitude that breast cancer equals death and lack of knowledge of early detection and screening techniques. Women felt that public education campaigns must be implemented to improve early detection and screening methods for breast cancer. The majority of beliefs regarding breast cancer and screening were common among urban and rural women.
Breast cancer treatment and sexual dysfunction:	Sbitti, Y.; Kadiri, H.; Essaidi, I.; Fadoukhair, Z.;	2011	BMJ	Questionnaire	120 Moroccn women with BC	University Military Hospital, Morocco	100% of participants have never spoken with their doctor about this subject. 84% of the participants continued sexual activity after treatment, but there was an increase in the incidence of sexual functioning

Supplementary 1. The included studies

Moroccan women's perception	Kharmoun, S.; Slimani, K.; Ismaili, N.; Ichou, M.; Errihani, H						problems which resulted in a slight reduction in the quality of their sex lives.
Social Support and Hope Among Egyptian Women with Breast Cancer after Mastectomy	Denewer, A.; Farouk, O.; Mostafa, W.; Elshamy, K.	2011	Breast Cancer: Basic and Clinical Research	Questionnaire	301 Egyptian women newly diagnosed with BC	Egypt	A low degree of hope was reported in 34.2% of patients and moderate degree in 36.2% and high degree in 29.6%.<br>A low degree of social support was reported in 39.5% and moderate degree n 33.6% and high degree in 26.9%.
Knowledge and Awareness of breast cancer among university female students in Muscat, Sultanate of Oman-A pilot study	Al Junaibi, R.M.; Khan, S.A.	2011	Journal of Applied Pharmaceutical science	Cross sectional Pilot study	157 female university students	Oman	The study results indicated that female students were well informed and aware about breast cancer in general, but their knowledge of breast cancer symptoms was better than the risk factors of breast cancer. The study also revealed that majority of the students knew that Breast self-examination (BSE) is the most common and easy method of breast cancer detection but their knowledge regarding frequency and the appropriate time to practice BSE was not very good.
Study exploring breast cancer screening practices amongst Arabic women living in the State of Qatar	Donnelly, T.T.; Al-Khater, A.-H.; Al-Kuwari, M.; Al-Meer, N.; Al-Bader,	2011	Avicenna<br><br>A Qatar foundation academic journal	Review	Arabic women residing in Qatar	Qatar	Even though it is challenging to compare figures between countries as each country's screening program is distinct, low breast cancer screening participation rate in Qatar should raise great concern. t times religion seems to work as an enabler in that it promotes internal locus of control when women believe that religion is urging them to take responsibility for their own health Although data

Supplementary 1. The included studies

	S.B.; Malik, M.; Singh, R.; Jong, F.C.						investigating these barriers and enablers in the Middle East, and specifically in Qatar, is scarce, the women's level of knowledge regarding breast cancer and its screening has been found to be related to screening behavior; lack of knowledge has been reported to act as a barrier for women to participate in screening activities . Studies show that level of knowledge is related to socio-economic status . Which in turn is related to mammography use . Furthermore, physician's recommendations have been found to be an enabler and likewise not receiving a recommendation has been found to be a barrier Other factors that act as barriers are fear of cancer, fear of finding out one has cancer, the notion that there is no cure, perceived benefit, time, cost, fear of gossip, fear that breast examination and mammography could be painful, husband or other male family members objecting to breast examination, preference for a female health professional, the health system, accessibility of the health system, perceived effectiveness and embarrassment
Experience of Syrian Women with breast cancer regarding chemotherapy: A qualitative study.	Nizamli, F.; Anoosheh, M.; Mohammadi, E.	2011	Nursing & Health Sciences	Semi-structured interviews	17 Women who underwent chemotherapy after mastectomy in Latakia Center	Syria	Four main themes emerged from the study: psychological discomfort (negative emotion, body image, and depressive symptoms), physical problems (acute consequences of chemotherapy and general aspects of chemotherapy), social dysfunction (social isolation and lack of marriage opportunities), and failure in the family role (mother role and sexual relationship).

Supplementary 1. The included studies

Knowledge, attitude & practice towards breast cancer & breast self-examination in Kirkuk University, Iraq	Alwan, N.A.S.; Al-Attar, W.M.; Eliessa, R.A.; Madfaie, Z.A.; Tawfeeq, F.N.	2012	Asian Pacific Journal of Reproduction	Pre-coded standardized questionnaire.	304 Educated Iraqi men and women at Kirkuk University	Kirkuk University, Iraq	It was observed that 177 (69.1%) of females have heard about the BSE. One hundred and nine of the female respondents (42.6%) reported that they have practiced BSE. Of those who have heard about BSE only 57.4% were in fact practicing the technique. Multiple logistic regression revealed that age of the respondents and knowledge on the means of early detection, the effect of nulliparity on the probability of contacting the disease and factors that could decrease the incidence of breast cancer were significantly associated with practicing BSE.<br>About half of the participants had a low knowledge score and only 14.3% were graded as "Good" and above. Almost 75% of the subjects believed that the best way to control breast cancer was through early detection.<br>90.9% of the subjects have heard about Breast self-examination and the main source of this information was the television.<br>Only 48.3% practiced Breast self- examination because of lack of knowledge of how to perform it correctly.
Voices of Fear and Safety" Women's ambivalence towards breast cancer and breast health: a qualitative study from Jordan.	Taha, H.; Al-Qutob, R.; Nyström, L.; Wahlström, R.; Berggren, V.	2012	BMC Women's Health	Explorative qualitative study	10 focus group. 65 healthy women	Jordan	Three themes were constructed from the group discussions: a) Ambivalence in prioritizing own health; b) Feeling fear of breast cancer; and c) Feeling safe from breast cancer. The first theme was seen in women's prioritizing children and family needs and in their experiencing family and social support towards seeking breast health care. The second theme was building on women's perception of breast cancer as an incurable disease associated with suffering and death, their fear of the risk of diminished femininity, husband's rejection and social stigmatization, adding to their apprehensions about breast health examinations. The third theme emerged from the women's perceiving themselves as not being in the

Supplementary 1. The included studies

							risk zone for breast cancer and in their accepting breast cancer as a test from God. In contrast, women also experienced comfort in acquiring breast health knowledge that soothed their fears and motivated them to seek early detection examinations.
Diagnosis delay in Libyan female breast cancer	Ermiah, E.; Abdalla, F.; Buhmeida, A.; Larbesh, E.; Pyrhönen, S.; Collan, Y.	2012	BMC Research Notes	Retrospective preclinical and clinical data were collected on a form (questionnaire) during an interview with each patient and from medical records.	200 women diagnosed between 2008 - 2009	Libya	Only 30.0% of patients were diagnosed within 3 months after symptoms. 14% of patients were diagnosed within 3-6 months and 56% within a period longer than 6 months. A number of factors predicted diagnosis delay: Symptoms were not considered serious in 27% of patients. Alternative therapy (therapy not associated with cancer) was applied in 13.0% of the patients. Fear and shame prevented the visit to the doctor in 10% and 4.5% of patients, respectively. Inappropriate reassurance that the lump was benign was an important reason for prolongation of the diagnosis time. Diagnosis delay was associated with initial breast symptom(s) that did not include a lump ($p < 0.0001$), with women who did not report monthly self examination ($p < 0.0001$), with old age ($p = 0.004$), with illiteracy ($p = 0.009$), with history of benign fibrocystic disease ($p = 0.029$) and with women who had used oral contraceptive pills longer than 5 years ($p = 0.043$). ($p < 0.0001$), and with metastatic disease ($p < 0.0001$).

Supplementary 1. The included studies

Age related quality of life among selected breast cancer patients in Aden, Yemen	Ba-Khubaira, S.; Al-Kahiry, W.	2012	Pan Arab Journal of Oncology	Cross sectional study using the FACT-B questioannaire	58 female patients	Yemen	The overall QOL score ranges from 40 to 134, with a mean of 77.6 (median 74). The mean score for various subscales were physical well being (GP) (21.3±3.2), social/family wellbeing (GS) (17.2±6.3), emotional wellbeing (GE) (18.2±2.1), functional wellbeing (GF) (7.8±2.7). The mean scores for breast cancer specific subscale (B) was 21.2 ± 5.4. Younger patients were having worse QOL in breast cancer specific concerns, except in B4 item (related to sexuality) when compared to older patients
Being within or being between? The cultural context of Arab women experience of coping with breast cancer in Israel.	Goldblatt, H.; Cohen, M.; Azaiza, F.; Manassa, R.	2012	Psycho-Oncology	Semi-structured interviews	20 Arab Muslim and Christian women with BC	Israel	Our main themes emerged: (i) “Family Comes First”: The Woman and Her Family; (ii) To Expose or not to Expose? That's the Dilemma; (iii) Faith in God as a Source of Coping; and (iv) Constructing New Meanings Following Recovery from Cancer. Findings stressed a duality in participants' cancer coping experience according to the traditional cultural norms of concealment, while simultaneously encountering more open Western attitudes through interactions with healthcare providers and Jewish women receiving treatment alongside them.
Health-related quality of life among breast cancer patients in Lebanon	Abu-Saad Huijer, H.; Abboud, S.	2012	European Journal of Oncology Nursing	Cross-sectional descriptive survey. Four instruments: European Organization for Research and treatment of Cancer-Quality of Life Questionnair	89 Lebanese women diagnosed with BC	Lebanon	The most prevalent symptoms were feeling nervous, feeling sad, lack of energy, and pain; symptom management was inadequate; high scores were reported on functional ability, medical care, spirituality, and relationships.<br>Payments per month for medical expenses, presence of metastasis, time since diagnosis, and type of treatment received were significantly associated with QoL, the different functioning dimensions, and symptoms.

Supplementary 1. The included studies

				e, Memorial Symptom Assessment Scale; Barthel Index; Needs at End of life Screening Tool, and a demographic/ clinical characteristics section.			
Breast cancer screening among Arabic women living in the State of Qatar: Awareness, knowledge, and participation in screening activities.	Donnelly, T.T.; Al Khater, A.-H.; Al-Bader, S.B.; Al Kuwari, M.G.; Al-Meer, N.; Malik, M.; Singh, R.; Chaudhry, S.; Dorri, R.A.	2012	Avicenna	quantitative, cross-sectional interview survey	1,063 Arabic women living in Qatar	Qatar	Of the 1,063 women interviewed (87.5% response rate), 90.7% were aware of breast cancer; 7.6% were assessed with having basic knowledge of BCS, 28.9% were aware of breast self-examination (BSE), 41.8% were aware of clinical breast exams (CBE), and 26.9% were aware of mammograms. Of the women interviewed, 13.8% performed BSE monthly, 31.3% had a CBE once a year or once every two years, and 26.9% of women 40 years of age or older had a mammogram once a year or once every two years. Participation rates in BCS activities were significantly related to awareness and knowledge of BCS, education levels, and receiving information about breast cancer, self-examination, or mammography from any of a variety of sources, particularly physicians.

Supplementary 1. The included studies

Beliefs and attitudes about breast cancer and screening practices among Arab women living in Qatar: A cross-sectional study	Donnelly, T.T.; Al Khater, A.H.; Al-Bader, S.B.; Al Kuwari, M.G.; Al-Meer, N.; Malik, M.; Singh, R.; Chaudhry, S.; Fung, T.	2013	BMC Women's Health	Multicentre, cross-sectional quantitative survey	1,063 arabic women	Qatar	In addition to low levels of awareness and low participation rates in BCS, one quarter of the participants stated their doctors talked to them about breast cancer, and less than half of the women interviewed believed breast cancer can be prevented. Women who engaged in BCS practice were more likely to have a doctor who talked to them about breast cancer, to believe they were in good-excellent health, that cancer can be prevented, or that cancer might be hereditary. The majority wanted to know if they had cancer and felt their health care needs were being met. The main reasons given for not planning BCS were lack of a doctor's recommendation, fear, and embarrassment.
Predictors of Quality of Life in a Sample of Lebanese Patients with Cancer	Abu-Saad Huijer, H.; Abboud, S.	2013	Europe's Journal of Psychology	A cross-sectional descriptive survey	200 Lebanese women and men with cancer	Lebanon	In the second model, the various symptoms of the EORTC QLQ-C30 were included and they explained 38.8% of the variance; the significant predictors were fatigue ($p = 0.00$) and appetite loss ($p = 0.03$) predicting poorer QoL

Supplementary 1. The included studies

Evaluation of Health Education on Knowledge , Attitude , Believes and Practice among Female Clerks as Regard Breast Self - Examination in Faculty of Medicine , Zagazig University , Egypt	El Badawy, A.A.; Eassa, S.; Koura, S.K.; Mohamed, H.A.M.	2013	The Egyptian Journal of Community Medicine	Intervention study	150 female clerks	Egypt, ZagaZig	The Health Education was effective in improving knowledge, attitude, and practice about BSE. Response of interviewed clerks about the different HBM items were significantly higher between pre, post and follow-up level except as regards their belief of the seriousness of breast cancer
Awareness of Breast Cancer among Female Students at Ain Shams University, Egypt	Boulos, D.N.K.; Ghali, R.R.	2013	Global Journal of Health Science	Descriptive cross-sectional study	543 female students from non-health related majors	Egypt	Most study participants had low level of knowledge of breast cancer risk factors. The most widely known risk factors by the students were smoking 66.9%, followed by radiation to the chest 63.7% and genetic factors 63.7%. Most of the students (81.6%) identified breast lump as a symptom for breast cancer. However, non-lump symptoms were less known and less than half were aware of other warning signs. Mass media such as TV and/or radio were identified as the main source of information on breast cancer by 89.1% of students followed by relatives 39.2%. Only 8.8% of students correctly identified the appropriate time to perform breast self examination and 1.3% reported performing it regularly every month. The most common reasons for not practicing BSE were" did not know how to perform it" (47.7%) and lack of interest (35%).
Quality of life of Bahraini women with breast cancer: a cross sectional study	Jassim, G.A.; Whitford, D.L.	2013	BMC Cancer	cross sectional study in which the European Organization for Research and	239 Bahraini women with BC	Bahrain	Among functional scales, social functioning scored the highest (Mean 77.5 [95% CI 73.65-81.38]) whereas emotional functioning scored the lowest (63.4 [95% CI 59.12-67.71]). The most distressing symptom on the symptom scales was fatigability (Mean 35.2 [95% CI 31.38-39.18]). Using the disease specific tool it was found that sexual functioning scored the lowest (Mean 25.9 [95% CI 70.23-77.90]). On the symptom

Supplementary 1. The included studies

				Treatment of Cancer Quality of Life Cancer Specific version translated into Arabic			scale, upset due to hair loss scored the highest (Mean 46.3 [95% CI 37.82-54.84]). Significant mean differences were noted for many functional and symptom scales.
Impact of maternal breast cancer on school-aged children in Saudi Arabia	Al-Zaben, F.; Al-Amoudi, S.M.; El-Deek, B.S.; Koenig, H.G.	2014	BMC Research Notes	39-item questionnaire face-to-face	28 women with BC	KSA	With regard to the way their children treated them, in 84% of cases mothers indicated that there was a positive change (i.e., a change for the better). In only two cases did mothers say the change was negative, one indicating that the child became angry and the other that the child withdrew from her. With regard to changes in the child's personality, in 90% of cases the change was reported to be positive and in only 1% (1 of 99 children) was it negative, i.e., the child became more emotional. Although in the vast majority of cases there was improvement in the way the child treated the mother, in the personality of the child, and in the relationship between mother and child, school performance did appear to suffer. In over three-quarters (77%), school performance worsened, whereas in only 8% did it improve.
What do Omani Women know about Breast Cancer Symptoms?	Renganathan, L.; Ramasubramaniam, S.; Al-Touby, S.; Seshan, V.; Al-Balushi, A.; Al-Amri, W.; Al-	2014	Oman Medical Journal	Cross sectional study	369 women	Oman	Among the total number of women 68 (19%) were calculated to have poor knowledge, 219 (59%) had average knowledge, 77 (21%) had good knowledge, and five (1%) had excellent knowledge on breast cancer. Among the variables, education status (p=0.002, p<0.050), and family history of breast cancer (p =0.000, p<0.010) was significantly related to a higher knowledge level.

Supplementary 1. The included studies

	Nasseri, Y.; Al-Rawahi, Y.						
Breast Cancer Screening Awareness, Knowledge, and Practice among Arab Women in the United Arab Emirates: A Cross-Sectional Survey	Elobaid, Y.E.; Aw, T.C.; Grivna, M.; Nagelkerke, N.	2014	PLoS ONE	Cross sectional survey + interviews	247 women	UAE, Al Ain	Despite the increase in the uptake of screening modalities in our study group, a lack of knowledge about breast cancer screening is still evident. Almost half (44.8%) of women who never had a Clinical Breast Exam (CBE) and 44.1% of women who never had a mammography expressed a lack of knowledge about the existence of these screening techniques. It was discovered that only 5% (n = 12) had a good general knowledge of breast cancer. UAE national women scored better than non-UAE national women younger women (40–49) had better scores regarding knowledge than older women (.49) The level of education was positively associated with better knowledge
Awareness and attitudes regarding breast cancer and breast self-examination among female Jordanian students	Suleiman, A.	2014	Journal of Basic and Clinical Pharmacy	Cross sectional questionnaire	900 female students	Jordan, university of Jordan in Amman	Approximately half of the respondents 435 (51.8%) were aware of breast cancer. Of these, 99 (22.7%) believed that it was caused by a medical condition, followed by old age (71; 16.4%), lack of breastfeeding (58; 13.3%), heredity (56; 12.8%), late marriage (44; 10.3%), pregnancies in older women (33; 7.5%), the use of brassieres (18; 4.1%), excessive breastfeeding (17; 3.9%), being unmarried (14; 3.2%), and spirituality (11; 2.6%). Overall, 152 participants (34.9%) were aware of BSE, but only 93 (11%) had performed it.

Supplementary 1. The included studies

Coping with a diagnosis of breast cancer amongst Omani women	Al-Azri, M.H.; Al-Awisi, H.; Al-Rasbi, S.; Al-Moundhri, M.	2014	Journal of Health Psychology	Semi-structured interviews	19 Omani women diagnosed with BC	Oman	Several coping strategies were identified including denial, optimism, withdrawal, Islamic beliefs and practices, and the support of family members and health-care providers, but Islamic beliefs and practices were the commonest.
Understanding the experiences and quality of life issues of Bahraini women with breast cancer	Jassim, G.A.; Whitford, D.L.	2014	Social Science & Medicine	Semi-structured interviews	12 Bahraini women with BC	Bahrain	The themes identified were meaning of cancer and quality of life, spirituality, and beliefs about causes of breast cancer, coping mechanisms, impact of illness and change in relationships. Quality of life was framed in terms of the ability to perform daily duties with emphasis on the physical component of quality of life. Themes that differed from previous western studies included a heavy emphasis on spiritual practices for comfort; the use of traditional clothing (hijab and abaya) to hide hair and body changes; the important role played by the family and husband in treatment decisions and concerns regarding satisfying the sexual needs of the husband, which were related to a fear of losing the husband to a second wife. Evil eye, stress and God's punishment were believed to be fundamental causes of the disease. The emotional shock of the initial diagnosis, concerns about whether to reveal the diagnosis and a desire to live a normal life were consistent with previous studies. However, cultural, and religious issues such as role of the husband and impact of prayers were also important

Supplementary 1. The included studies

							here.
Knowledge, Attitude and Practice of Breast Cancer Screening among Female General Practitioners in Riyadh, Saudi Arabia.	Saeedi, M.	2014	Cancer Research Journal	Cross sectional	Female family physicians		The majority (90%) of the respondents believed that Breast Cancer is a major health problem and 96% were aware of the importance of mammography as a screening method. But only 19% of the studied physicians requested mammogram as screening tool for women aged 40 years or more
Factors that Influence Awareness of Breast Cancer Screening among Arab Women in Qatar: Results from a Cross Sectional Survey	Donnelly, T.T.; Al Khater, A.H.; Al-Bader, S.B.; Al Kuwari, M.G.; Malik, M.; Al-Meer, N.; Singh, R.; Fung, T.	2014	Asian Pacific Journal of Cancer Prevention	A multicenter cross-sectional survey`	1,063 Arabic women	Qatar	While most participants (90.7%) were aware of breast cancer, less than half had awareness of BCS practices (28.9% were aware of breast self-examination and 41.8% of clinical breast exams, while 26.4% knew that mammography was recommended by national screening guidelines. Only 7.6% had knowledge of all three BCS activities). Regarding BCS practice, less than one-third practiced BCS appropriately (13.9% of participants performed breast self-examination (BSE) monthly, 31.3% had a clinical breast exam (CBE) once a year or once every two years, and 26.9% of women 40 years of age or older had a mammogram once every year or two years). Awareness of BCS was

Supplementary 1. The included studies

							significantly related to BCS practice, education level, and receipt of information about breast cancer and/or BCS from a variety of sources, particularly doctors and the media.
Quality of life and psychological well-being of breast cancer survivors in Jordan	Abu-Helalah, M.A.; Alshraideh, H.A.; Al-Hanaqta, M.M.; Arqoub, K.H.	2014	Asian Pacific journal of cancer prevention	Cross sectional study	236 psychological aspect for breast cancer survivors in Jordan	Jordan	Among functional scales, "social functioning" scored the highest (mean=78.1±28.6 SD), whereas "emotional functioning" scored the lowest. For the QLQ-BR23, the worst scores within the functional scales were for "body image" and "future perspective". The worst symptom was "upset by hair loss". 53% scored abnormal on the anxiety scale and 45% on the depression scale. Severe depression and severe anxiety were detected among 8% and 14% of study participants, family history of cancer, low educational status, current social problems, extent of the disease, presence of financial difficulties, and employment status.
Psychosocial Impact of Breast Cancer Diagnosis Among Omani Women.	Al-Azri, M.; Al-Awisi, H.; Al-Rasbi, S.; El-Shafie, K.; Al-Hinai, M.; Al-Habsi, H.; Al-Moundhri, M.	2014	Oman medical journal	Cross-sectional semi-structured interview	19 Omani women with BC	Oman, Muscat	Four main themes emerged. These were: a) factors related to psychological distress of the disease and uncertainty (worry of death, interference with work and family responsibilities, searching for hope/cure, travelling overseas); b) reactions of family members (shocked, saddened, unity, pressure to seek traditional treatments); c) views of society (sympathy, isolation, reluctant to disclose information); and d) worries and threats about the future (side effects of chemotherapy, spread of the disease, effect on offspring).<br>The availability of such services in their home country could also help reduce anxiety from being away and they could get support from family members. Some of the participants felt that the relationships with their family members, including husbands, became stronger after diagnosis and that family members became more supportive

Supplementary 1. The included studies

Do socioeconomic factors influence breast cancer screening practices among Arab women in Qatar?	Donnelly, T.T.; Al Khater, A.H.; Al Kuwari, M.G.; Al-Bader, S.B.; Al-Meer, N.; Abdulmalik, M.; Singh, R.; Chaudhry, S.; Fung, T.	2015	BMJ Open	Multicenter cross-sectional study	1,063 Arabic women	Qatar	Findings indicate that less than one-third of the participants practiced BCS appropriately, whereas less than half of the participants were familiar with recent BCS guidelines. Married women and women with higher education and income levels were significantly more likely to be aware of and to practice BCS than women who had lower education and income levels.
Complementary and alternative medicine use and its association with quality of life among Lebanese breast cancer patients: A cross-sectional study.	Naja, F.; Fadel, R.A.; Alameddine, M.; Aridi, Y.; Zarif, A.; Hariri, D.; Mugharbel, A.; Khalil, M.; Nahleh, Z.; Tfayli, A.	2015	BMC Complementary Alternative Medicine	Cross sectional study done in 2 major referral centers	180 women with BC	Lebanon	Among study participants recruited from both sites, the most commonly used CAM was 'special food' followed by 'herbal teas', 'diet supplements' and 'Spiritual healing'. Only 4 % of CAM users cited health professionals as influencing their choice of CAM and only one in four patients disclosed CAM use to their treating physician. There was no significant association between CAM use and QOL.
Using the health belief model to predict breast self-examination among Saudi women.	Abolfotouh, M.A.; BaniMustafa, A.A.; Mahfouz, A.A.; Al-Assiri, M.H.; Al-Juhani, A.F.;	2015	BMC Public Health	Cross sectional: Arabic version of revised Champion's Health Belief Model Scale (CHBMS) and the Arabic version of	225 Saudi female employees, working at King Abdulaziz Medical City and their non-working adult	KSA, Riyadh	The majority of women heard about BSE (91.2 %), only 41.6 % reported ever practicing BSE and 21 % performed it regularly. Reported reasons for not doing BSE were not knowing how to examine their breast (54.9 %), or untrusting themselves able to do it (24.5 %). Women were less knowledgeable about BC in general, its risk factors, warning signs, nature, and screening measures (PMS:54.2 %, 44.5 %, 61.4 %, 53.2 %, 57.6 % respectively). They reported low scores of; perceived susceptibility, seriousness,

Supplementary 1. The included studies

	Alaskar, A.S.			Breast Cancer Awareness Measure (CAM),	female family member = 208		confidence and barriers (PMS: 44.8 %, 55.6 %, 56.5 % & 41.7 % respectively), and high scores of perceived benefits and motivation (PMS: 73 % & 73.2 % respectively) to perform BSE. Significant predictors of BSE performance were: levels of perceived barriers (p = 0.046) and perceived confidence (p = 0.001) to BSE, overall knowledge on BC (p < 0.001), work status (p = 0.032) and family history of BC (p = 0.011).
A cross-sectional study of anxiety and marital quality among women with breast cancer at a university clinic in western Saudi Arabia	Al-Zaben, F.N.; Sehlo, M.G.; Koenig, H.G.	2015	Saudi Medical Journal	participants completed the Hospital Anxiety and Depression Scale, Spouse Perception Scale, and Quality of Marriage Index forms, and answered questions on demographic and cancer characteristics.	49 married women with BC	KSA	Anxiety symptoms indicating "possible" anxiety disorder were present in 10.4% and "probable" anxiety disorder in 14.6% (25% total). No significant relationship was found between the quality of marital relationship and anxiety symptoms (B=-0.04, standard error=0.05, t=-0.81, p=0.42). Anxiety was primarily driven by low education, poor socioeconomic status, and young age.
A cross-sectional assessment of quality of life of breast cancer patients in Saudi Arabia.	Almutairi, K.M.; Mansour, E.A.; Vinluan, J.M.	2016	Public Health	Cross sectional study. Arabic version of EORTC QoL questionnaire	145 female cancer patients	KSA, Riyadh	The most distressing symptom on the symptom scale was insomnia (mean 84.14 [95% CI 79.95-88.32]), followed by appetite loss (mean 80.92 [95% CI 76.51-85.33]) and dyspnoea (mean 80.00 [95% CI 75.51-84.49]). Poor functioning was found in sexual enjoyment (mean 22.52 [95% CI 17.97-27.08]) while future perspective scored the highest (mean 76.32 [95% CI 70.52-82.12]).

Supplementary 1. The included studies

Breast self-examination (BSE): Knowledge and practice among female faculty of physical education in Assuit, South Egypt	Abou, H.; Bayumi, E. azayiem	2016	Journal of Medicine, Physiology and Biophysics	Self-administered questionnaire	240 students	Egypt	More than half of the students were a poor knowledge about breast cancer risk factors and presentations, only (20.8%) had a good knowledge. It was reported that (75%) of them she know that the exposure to radiation is a main risk factor. University female students had insufficient knowledge about breast self-examination and the main source of information was the media as reported by (36.7%) of the students, mentioned that breast self-examination should be practiced monthly, (57.9%) of them knew the right way to carry out breast self-examination.. There was a significant relation between breast self-examination practice and knowledge about breast cancer and knowledge about breast self- examination.
Breast cancer presentation delays among Arab and national women in the UAE: A qualitative study	Elobaid, Y.; Aw, T.C.; Lim, J.N.W.; Hamid, S.; Grivna, M.	2016	SSM Popul Health	Semi-structured interviews	19 BC survivors	UAE	Some of the women did not know about relevant BC symptoms and signs, screening procedures and treatment options.<br>The participants did not view themselves as at risk for BC. Education level did not appear to influence their behavior in regards to measures for early detection of BC. Many felt that they had to fulfill multiple roles in regards to their families and to society. They felt pressure because they were expected to be role models in their society and cancer is thought to be affecting this role, and the negative attitude of community members to them once they developed the disease. Most of the women shared the view that cancer was not a popular topic to talk about in their society or even within the family. It is a sensitive topic surrounded by shame and silence.
Health-Related Quality of Life among Breast Cancer Patients and Influencing Factors in Morocco	El Fakir, S.; El Rhazi, K.; Zidouh, A.; Bennani,	2016	Asian Pacific Journal of Cancer prevention	Prospective study using Moroccan Arabic versions of the European	1,463 women with BC	Morocco	A total of 1463 subjects were included in the study, with a mean age of 55.6 (SD. 11.2) years, 70% being married. The majority had stage II (45.9%) and a few cases stage IV (12.9%) lesions. The participants' global health mean score was 68.5 and in "functional scales", social functioning scored the highest (Mean

Supplementary 1. The included studies

	M.; Benider, A.; Errihani, H.; Mellass, N.; Bekkali, R.; Nejjari, C.			Organization for Research and Treatment of Cancer Quality of Life Questionnair e C 30 (EORTC QLQ C30) and the Breast Cancer-Specific Quality of Life Questionnair e (EORTC QLQ-BR23).			86.2 (SD=22.7)). The most distressing symptom on the symptom scale was financial difficulties (Mean 63.2 (SD=38.2)). Using the disease specific tool, it was found that future perspective scored the lowest (Mean 40.5 (SD=37.3)). On the symptom scale, arm symptoms scored the highest (Mean 23.6 (SD=21.6)). Significant mean differences were noted for many functional and symptom scales.
Knowledge and practice of university female students toward breast cancer.	l-Haddad, M.; Al-Adwani, M.; Abu-Rukbah, W.; Al-Otaibi, A.; Al-Hayfani, M.	2016	Archives of Pharmacy Practice	Cross sectional survey of 6 parts	826 female students	KSA, Taif University.	Majority of respondents showed low level of knowledge regarding breast cancer. The Internet was the main source of knowledge 53.5%. Moderate knowledge regarding breast cancer symptoms were observed by more than 50% of respondents. Knowledge regarding risk factors was low by majority of respondents >50%. In addition, only 28% of respondents practice breast self-examination (BSE), whereas main barrier for not practicing BSE was the lack of knowledge on how to perform BSE 57.5%.
Study of quality of life and characteristic factors in women with breast cancer	Al-Naggar, R.; Osman, M.; Al-	2016	J Appl Pharm Sci	Cross sectional study using The Functional Assessment	166 women with BC	Iraq, Baghdad	.The study found a significant difference between the quality of life among cancer patients and job, stage of cancer, size of tumor, and radiotherapy. Job, stage of cancer, size of tumor, and radiotherapy significantly influenced the women QoL.

Supplementary 1. The included studies

undergoing different types of therapy	Baghdadi, N.			of Cancer Therapy-Breast (FACT-B)".			However, there was a significant difference between the quality of cancer patients and radiotherapy
The predictors of poor quality of life in a sample of Saudi women with breast cancer.	Ahmed, A.E.; Alharbi, A.G.; Alsadhan, M.A.; Almuzaini, A.S.; Almuzaini, H.S.; Ali, Y.Z.; Jazieh, A.R.	2017	Breast Cancer: Targets and Therapy	Cross-sectional study	145 Saudi women with BC	KSA	We noted worse bodily pain in breast cancer patients with late-stage cancer (III or IV), multiple tumors, metastasis, and fever. We discovered poor general health in patients with multiple tumors, metastasis, and fever. Breast cancer patients who received immunotherapy were predicted to have less vitality and increased pain by scores of 7.2 and 10.8, respectively. Multiple tumors were predicted to worsen role limitations due to emotional problems and social function by scores of 31.6 and 20.6, respectively. Newly diagnosed breast cancer patients with first-year-after-diagnosis tended to have a decrease in social function by a score of 14.2
Anxiety, Depression and Quality of Life in Breast Cancer Patients in the Levant	Akel, R.; El Darsa, H.; Anouti, B.; Mukherji, D.; Temraz, S.; Raslan, R.; Tfayli, A.; Assi, H.	2017	Asian Pacific Journal for Cancer Prevention	Cross sectional study using the Hospital Anxiety and Depression Scale (HADS) and the Functional Assessment of Cancer Therapy-Breast (FACT-B)	150 female women patients with BC	Lebanon	Most were assessed 3 to 5 years (68.7%) after initial diagnosis and had undergone surgery, chemotherapy, radiation, or hormonal therapy (97.3%, 79.3%, 80.7% and 86.0%, respectively). The median total HADS score was 10.0 ± 8.0, with approximately 41.3% of study participants having abnormal scores on the anxiety subscale and 24.7% on the depression subscale. Significant predictors of total HADS score were nationality and level of education (p=0.001, p=0.001 respectively; R2=0.181). Participants who were Iraqi, had stage IV disease, had a household monthly income below 1000 USD, or had received chemotherapy exhibited significantly lower total FACT-B scores, these being highly negatively correlated with total HADS scores (rs= -0.73, p=0.001).
Knowledge and Perceptions of Common Breast	Alrashidi, A.G.; Ahmed,	2017	Asian Pacific Journal for	Cross sectional study	165 males and 401	KSA	With regard to breast cancer risk factors, 427/566 (75.4%) of participants answered in the affirmative to whether breast cancer could be inherited. For early

Supplementary 1. The included studies

Cancer Risk Factors in Northern Saudi Arabia	H.G.; Alshammeri, K.J.K.; Alrashedi, S.A.; ALmutlaq, B.A.; Alshammari, F.N.M.; Alshudayyid, A.A.H.; Alshammari, A.A.J.; Anazi, F.M.S.; Alshammari, W.M.; et al.		Cancer Prevention		females all Saudi		puberty and late menopause, 209/566 (37%) were in agreement with increased risk, for low and delayed child birth, 261/566 (46%), and for overweight and obesity, 210/566(37%). For the question of whether natural breast feeding can reduce the risk of breast cancer, only 35/566 (6.2%) said yes.
From a Death Sentence to a Disrupted Life.	Hammoudeh, W.; Hogan, D.; Giacaman, R.	2017	Qualitative Health Research	Semi-structured interviews	35 Palestinian women with BC	Palestine	Three themes emerged: (a) the transition from initial shock to the daily struggles with disruptions caused by illness, (b) the role of social support in helping women cope with the burden of disease, and the importance of (c) faith and reliance on God (tawakkul). In the Palestinian context, women's narratives highlighted the disruptive nature of breast cancer.
Patterns and determinants of mammography screening in Lebanese women.	Elias, N.; Bou-Orm, I.R.; Adib, S.M.	2017	Preventive medicine reports	Cross sectional survey	2400 Lebanese women	Lebanon	Of the total, 105 women (4·4%) had never heard of mammography as a tool for early breast cancer detection. Among the remaining 2295, 45% had ever used it, of whom 10% had obtained it for the first time within the 12 months preceding the survey. Repeaters were 67% of 926 women who had the time opportunity to do so (median lifetime frequency: 2). Older age, higher socio-economic status (SES) and living within the Greater Beirut (GB) area were significantly associated with ever-use. Within

Supplementary 1. The included studies

							GB, psychosocial factors such as perceived susceptibility and benefits were most strongly associated with ever-use. Outside GB, socio-economic advantage seemed to mostly affect ever-use. Only 4% reported opposition from husbands to their mammography, and husband's support was significant for adherence to mammography guidelines mostly outside GB. Higher education emerged also as a significant socio-demographic determinant for ever-repeating in all regions. Perceived comfort of the previous test strongly affected the likelihood of repeating it.
Cancer-related post-treatment pain and its impact on health-related quality of life in breast cancer patients: A cross sectional study in Palestine	Abu Farha, N.H.; Khatib, M.T.; Salameh, H.; Zyoud, S.H.	2017	Asia Pacific Family Medicine	Correlational Cross-sectional study	170 women with BC	Palestine	Furthermore, univariate analysis showed that age, marital status, employment status, income, current condition of cancer, and post-treatment pain were associated with quality of life (p-value < 0.05). Regression analysis revealed that patients with high income (p-value = 0.003), patients with lower pain severity score (p-value < 0.001), and lower pain interference score (p-value = 0.018) were independently associated with high QOL.
The impact of Breast Cancer on quality of life among a sample of female Iraqi patients.	Daher, A.M.; Al-Rubai, T.A.; Al-Nuaimi, A.S.; Al-Shawi, A.F.; Medhat, U.	2017	International Journal for Quality Research	Cross-sectional questionnaire	263 patients with BC	Iraq	The highest negative impact was for Breast Cancer impact on happiness, followed by "ability to focus on daily tasks". Sleep was the third negatively affected domain of life and "carrying out house chores" was the fourth and last domain where more than half of participants showed a negative impact. There was a high percentage of observed agreement between the calculated breast cancer impact and the perceived overall negative impact of breast cancer assessed by direct questioning. This high observed agreement was significantly beyond chance.
The Relationship Between Spirituality and Quality of Life	Al-Natour, A.; Al	2017	Journal of Religion and Health	Descriptive cross-sectional	150 Jordanian	Jordan	Among the four domains of the QoL, the social/family well-being domain reported the highest score with

Supplementary 1. The included studies

of Jordanian Women Diagnosed with Breast Cancer.	Momani, S.M.; Qandil, A.M.A.			Arabic version of the Functional Assessment of Chronic Illness Therapy-Spiritual Well-being	women with BC		mean 23.21 (SD = 5.37), while the lowest was physical well-being with a mean 18.57 (SD = 6.93). A positive linear relationship was found between spirituality and QoL, r = 0.67, p = 0.000. The highest score of correlation was seen between spirituality and the functional domain, r = 0.63, p = 0.000. Spiritual intervention could contribute to increased functional, social, and physical well-being and enhance the total health and QoL of women diagnosed with breast cancer.
Isolation and prayer as mean of solace for Arab women with breast cancer: An in-depth interview study	Assaf, G.N.; Holroyd, E.; Lopez, V.	2017	Psycho-Oncology	Face-to-face depth interviews	20 Arab women attending a public hospital in Abu Dhabi	UAE	Arab women's experiences following their breast cancer diagnoses and treatments included the themes of (1) protecting one's self from stigma, (2) facing uncertainties and prayers, and (3) getting on with life. Overall, the ways to find solace were through isolation and prayer, which are heavily influenced by religion and spiritual practices. They recommended that to help women with breast cancer, a campaign to raise awareness for early screening is needed as well the need to form a peer-led support group for women with breast cancer consisting of breast cancer survivors so that they can learn from each other's experiences.
Patterns, knowledge and barriers of mammography use among women in Saudi Arabia	l-Wassia, R.K.; Farsi, N.J.; Merdad, L.A.; Haj, S.	2017	Saudi Medical Journal	Cross-sectional survey	3,245 women older than 40 years	KSA	Approximately 36% of the women perceived their knowledge as poor; 24% perceived their knowledge as excellent, 22% perceived their knowledge as fair and 19% of the respondents perceived their knowledge as very good. The main reasons why women failed to obtain a mammogram were the belief that the examination was not important (31%) and worries about the results (25%). Regarding how participants would feel before a mammography appointment, 45.5% reported they would be worried about the results, 15.7% reported they would be scared of pain, 11.1% reported they would not be able to sleep, 10.3% reported that they

Supplementary 1. The included studies

							would be embarrassed, and 7.5% reported that they would not want to go
Depression and Anxiety among Females with Breast Cancer in Sohag University: Results of an Interview Study	Aly, H.; ElGhany, A.; Abd ElLateef, A.; El Sayed Mohamed, A.	2017	Remedy Open Access	Cross sectional observational study	96 women with BC	Egypt, Sohag	One-third reported an advanced degree of depression, anxiety or both (33.34%; 33.33%, and 32.29%, respectively). As regards associations; patients with progressive/relapse disease have higher anxiety and depression with sustained statistically significant relationship in univariate and multiple regression analyses (p-value = 0.03 and 0.04, respectively); while hormonal treatment has a statistically significant positive impact on anxiety alone (p-value 0.02).
Sociocultural Influences on Arab Women's Participation in Breast Cancer Screening in Qatar.	Hwang, J.J.; Donnelly, T.T.; Ewashen, C.; McKiel, E.; Raffin, S.; Kinch, J.	2017	Qualitative Health Research	Semi-structured interviews	15 health practitioners	Qatar	Through thematic analysis of the data, we found three major factors influencing breast cancer screening practices: (a) beliefs, attitudes, and practices regarding women's bodies, health, and illness; (b) religious beliefs and a culturally sensitive health care structure; and (c) culturally specific gender relations and roles
Knowledge and practice of breast self-examination among sample of women in Shatra/Dhi-Qar/Iraq	Ewaid, S.H.; Shanjar, A.M.; Mahdi, R.H.	2018	Alexandria Journal of Medicine,	Cross-sectional survey	200 participants	Iraq	Only 25.4% students, 24.4% teaching staff, and 21.2% employees actually practiced BSE.<br>Fifty-four percent of students, 42% teaching staff, and 37% employees know that a mammography is a tool for screening of BC. Most of the participants had knowledge about warning signs of BC but only half of all the participants know that the best time for BSE is 5th to 7th day after menstrual cycle and only 31% know that the correct age to do BSE is >18 year age. TV and internet were the main sources of knowledge about SBE for 47% of all participants.
Quality of life and complementary and alternative medicine	Albabtain, H.; Alwhaibi,	2018	Saudi Pharma J	Cross-sectional study	95 women Saudi	KSA, Tertiary hospital	The most commonly used CAM therapy was spiritual therapy 70.5%, followed by honey 36.8%, olive oil 24.2% and 23.2% herbal therapy. With regards to

use among women with breast cancer.	M.; Alburaikan, K.; Asiri, Y.				women with BC		QoL, there was a statistically significant difference between CAM users and non-CAM users in global health status (73.2% vs. 64.8%, P = 0.049).
Assessment of Knowledge, Attitude and Breast Self-Examination Practice among Female Students in Sulaimani Polytechnic University	Mohammed	2018	Kurdistan Journal of Applied Research	Survey + Interview	216 female students from 4 different faculty	Iraq	he mean age of participants was 20.7, the study result shows that the knowledge mean score of participants was 3.95 (SD ± 1.78).181(83.8%) have information of breast cancer, and 157(72.7%) have information on breast cancer sign,136(63.0%) have information on factor that increase change to get breast cancer,150(69.4%)have information on (BSE),86(39.8%)have information how to do (BSE),101(46.8%)have information on mammography .91(42.1%)practice of (BSE), only 10 (4.6 %) female students has performing this examination regularly , percentage of students that did not done mammography was 193 (89.4%) . only 2 (0.9 %) performs mammography screening regularly, 18 (8.3 %) have a family member that get breast cancer.
Knowledge, Attitude and Practice of Breast Self-Examination Among Female	Haddad, L.	2018	EMPHNET 6th Regional Conference	Cross sectional survey	Governmental schoolteachers	Palestine	From what is shown, the majority of the teachers were aware of BSE with percentage 97,3% and 76,9% of them only knew the procedures of BSE and actually 47,6% of them do not make BSE periodically. The study also showed that 57.1% of the respondents knew about the breast cancer from the media, followed by 25, 2% from health workers; in addition, the study revealed 77.6% are aware about the symptoms of breast cancer, while 81.6% showed that is BSE is not enough to diagnose breast cancer.
Health-related quality of life: Impact of surgery and treatment modality in breast cancer	Darwish, D.; Enien, M.; Ibrahim, N.; Makar,	2018	. Journal of Cancer Research and Therapeutics	This is a cross-sectional study. The Arabic version of EORTC	172 Egyptian women with breast cancer	Egypt	Among the functional scales of QLQ-C30, social functioning scored the highest (87.91 ± 17.92, 95% CI: 91.64) whereas emotional functioning scored the lowest (59.61 ± 24.96, 95% CI: 64.66). The most distressing symptom on the symptom scales of QLQ-C30 was financial impact followed by fatigue and pain (mean: 57.87, 39.43, and 36.44). Using the

Supplementary 1. The included studies

	W.; Gaber, M.			QLQ-C30 (version 3) and EORTC QLQ-BR23 questionnaire			disease-specific tools, it was found that body image and sexual functioning scored the lowest (mean 74.51 ± 13.21 and 74.45 ± 14.89, 95% CI: 77.27 and 77.55), respectively. On the symptom scale, arm symptoms scored the highest with a mean of 32.35 ± 23.22 (95% CI: 37.19). MRM patients had more favorable global health status and body image among the functional scale (P = 0.011, 0.027) due to social and religious issues. The functional scale was better in BCS with significant role function (P = 0.004). In the symptom scale, fatigue, pain, systemic side effects, and arm symptoms were statistically significant better in the BCS (P = 0.004, 0.006, 0.002, and 0.003, respectively).
Knowledge, Attitudes, and Practices of Breast Cancer Screening Methods Among Female Patients in Primary Healthcare Centers in Najran, Saudi Arabia	Alshahrani, M., Alhammam, S., Al Munyif, H., Alwadei, A., Alwadei, A., Alzamanan, S. and Aljohani, N.	2018	Journal of Cancer Education	Cross sectional study: survey and direct interviews	500 females with BC	KSA, Najran PHCC	Nineteen percent of patients demonstrated a high knowledge of breast self-examination. Breast self-examination was performed by 35% of patients, whereas 15% of patients received mammograms and 19.8% clinical breast examinations. The most common barrier for screening methods of breast cancer was that patients were unaware of half of the screening methods. A total of 20.6% of women did not perform breast self-examinations because they were not well trained, and 26.4% of women did not receive clinical breast examinations because there was no female doctor available. The main source of information among the women patients was social media (52.4%), whereas 8.8% received information through their healthcare provider.
Determinants of breast cancer in Saudi women from Makkah region: a case-control study (breast cancer risk	Alsolami, F.J.; Azzeh, F.S.; Ghafouri, K.J.; Ghaith,	2019	BMC Public Health	Case- control study	432 Saudi females newly diagnosed with BC	KSA, Makkah	With regard to the socioeconomic factors, the results showed that being unemployed had an increased positive association with breast cancer (β = 1.89, OR = 6.56, 95% CI = 3.83–11.37, P < 0.001). This was similar to the results of being in the low-income category of < 5000 SR (~ 1333.17 USD) (β = 3.69, OR = 39.88, 95% CI = 11.11–143.16, P < 0.001)

factors among Saudi women)	M.M.; Almaimani, R.A.; Almasmoum, H.A.; Abdulal, R.H.; Abdulaal, W.H.; Jazar, A.S.; Tashtoush, S.H.						High percentages in both groups exhibited breastfeeding histories, with the results showing that most of the cases (70%) breastfed for a duration of 6–12 months, while most of the participants in the control group breastfed for a duration of less than 6 months
Knowledge, Attitude and Practice of Breast Self-Examination Among Sudanese Females Attended to Omdurman Maternity Hospital in Sudan	Kunna, A.; Ismail, K.; Enour, S.; Taha, U.; Elkheir, H.	2019	Ann Med & Surg Case Rep	prospective cross-sectional hospital-based study	389 females attending Gynecological and Obstetric departments at Omdurman Maternity Hospital	Sudan	The overall score of knowledge and attitude about breast self-examination were found moderate (54.6% and 59.8% respectively); among which knowledge and attitude were most commonly poor regarding time of performing the BSE and phobia of detecting the cancer (39.6% and 42.2% respectively). The study also revealed poor performance towards BSE (34.6%). Knowledge about BSE found significantly associated with family history of breast cancer, age and education (P< 0.05). Practicing BSE found significantly associated with higher level of education, receiving training or counseling and history of other breast disease (P<0.05). Media and heath cadres are the commonest source of knowledge about breast self-examination (41.5% and 21.3% respectively).
Quality of life of women with breast cancer undergoing treatment and follow-up at King Salman Armed	Al Zaharani, A.M.; Alalawi, Y.; Yagoub, U.; Saud,	2019	Breast cancer : Targets and Therapy	Cross-sectional study questionnaire	96 Saudi female patients with BC	KSA, Tabuk	The highest score was observed for the physical well-being subscale (7.65±071), followed by the spiritual well-being subscale (7.19±0.66), psychological well-being scale (7.09±0.30) and social concerns subscale (7.02±0.41). Age, marital status and cancer stage differed remarkably (P=0.00) for the physical well-being subscale scores. The social well-being subscale score of single and married women was significantly

Supplementary 1. The included studies

Forces Hospital in Tabuk, Saudi Arabia	N.; Siddig, K.						higher than that of widowed and divorced women. Women who underwent radiation and immunotherapy had significantly higher scores for the spiritual well-being subscale.
Awareness Level, Knowledge and Attitude towards Breast Cancer between Medical and Non-Medical University Students in Makkah Region: A Cross Sectional Study	Sindi, R.; Alzahrani, A.; Alzahrani, N.; Alshareef, S.; Tabassum, A.; Iqbal, M.; Salman, R.	2019	International Journal of Cancer and Clinical Research	Cross sectional close-ended questionnaire	250 female medical and 250 female non-medical students	KSA, Makkah	With regard to the participants' knowledge about BSE (Breast Self Examination) and mammography, the data showed that the majority of both medical and non-medical students were aware of the fact that BSE helps in the early detection of breast cancer. The participants in both groups of this study also had good knowledge about how to practice BSE. However, only 61% medical and 50% non-medical students performed BSE. On the other hand, only small proportion (24%) of the participants knew that periodic mammography is an important tool for early screening of breast cancer, and only 3% of students involved in this study confirmed having screened by mammogram. Among the proportion of participants who revealed that they have never performed BSE, about 57% of medical and 43% of non-medical students reported that there is no need to perform BSE. A good number of participants from both groups revealed that they do not know how to perform BSE, while very few in both groups reported discomfort as a reason behind the poor practice of BSE. Fear and shyness were the other reasons for not performing BSE reported by both medical and non-medical students respectively
Factors Affecting Health Related Quality of Life among Women with Breast Cancer Receiving Chemotherapy	Al Shaikh, S.; Sofar, S.	2019	American Journal of Nursing Research	Quantitative descriptive correlational design	122 women diagnosed with BC and receiving chemotherapy	KSA	More than half 51.6% of the studied women were moderately affected regarding overall factors affecting health related quality of life. There was a significant negative relationship between psychological factors and quality of life ($P < 0.001$).

Supplementary 1. The included studies

Country	Summary of the Main Findings
**Bahrain**	Emotional and sexual functioning scored the lowest and the main concern affecting quality of life was fatigability and hair loss. Evil eye, stress and God's punishment were believed to be fundamental causes of the disease. Factors associated with a major reduction in all domains of quality of life included the presence of metastases, having had a mastectomy as opposed to a lumpectomy
**Egypt**	Women tend not to visit the doctor unless they are severely ill which resulted in delayed diagnosis and low screening level. Women believed that they should directly visit a gynecologist in case of breast cancer and wanted doctors to be more encouraging. An interventional study for health education about BC was found to be effective. Female students had a poor knowledge related to BSE practices. 34.2% of women with BC had low hope and 32% reported having both depression and anxiety, therefore, emotional functioning, had the lowest scores.
**Iraq**	The majority of the subjects have heard about Breast self-examination and the main source of this information was the television and internet even though only 48.3% practiced BSE and 187 only had a mammogram in the past. Iraqi women who were diagnosed with BC, job, stage of cancer, size of tumor, and radiotherapy significantly influenced their QoL. The highest negative impact was for Breast Cancer impact on happiness, followed by "ability to focus on daily tasks". Sleep was the third negatively affected domain of life and "carrying out house chores" was the fourth and last domain where more than half of participants showed a negative impact.
**Jordan**	Women tend to prioritize their families, children and chores as mothers and wives over themselves. Women were also perceiving themselves as not being in the risk zone for breast cancer and in their accepting breast cancer as a test from God. Women with BC scored the lowest on emotional functioning due to the hair loss. Spiritual intervention could contribute to increased functional, social, and physical well-being and enhance the total health and QoL of women diagnosed with breast cancer.
**KSA**	Attendance of Saudi women to screening and CBE visits found to be low. This was associated with the low knowledge that women included in the studies had about the incidence of BC and its risk factors as well as lack of female doctors. Their main source of information was the media but they insisted on the need of recommendation from physicians. Level of knowledge regarding risk factors and appropriate screening was low and dependent upon educational and occupational status The most frequently reported risk factors were non-breast feeding and the use of female sex hormones. Barriers to not performing BSE were lack of confidence, anxiety, and not knowing how to. The most commonly used CAM therapy was spiritual therapy 70.5%.

Supplementary 1. The included studies

**Kuwait**	Despite institutional supports, Kuwaiti women had clinically significant poorer global QOL and functional scale scores, more intense symptom experience, in comparison with the international data. Younger women had poorer HRQOL scores
**Lebanon**	The utilization of mammography found to be low and increased only slightly over 4 years (from 11% to 18%). Payments per month for medical expenses, presence of metastasis, time since diagnosis, and type of treatment received were significantly associated with QoL, the different functioning dimensions, and symptoms. The most commonly used CAM was 'special food' followed by 'herbal teas', 'diet supplements' and 'Spiritual healing'. Only 4 % of CAM users cited health professionals as influencing their choice of CAM
**Libya**	Only 30.0% of patients were diagnosed within 3 months after symptoms. Main causes of delayed diagnosis of BC among Libyan females were: fear and shame about this sensitive topic, inappropriate reassurance that the lump was benign, symptoms were not considered to be serious.
**Morocco**	Female doctors were the only professional group that had satisfactory knowledge of risk factors of BC and there was a common belief that herbal medicine can cure BC. 100% of participants have never spoken with their doctor about this subject. 84% of the participants continued sexual activity after treatment, but there was an increase in the incidence of sexual functioning problems which resulted in a slight reduction in the quality of their sexual lives.
**Oman**	Female students had good knowledge on symptoms of BC and about BSE, but were not trained on how to perform it. Educational status and family history were indicative factors for the higher knowledge about BC. Omani women felt more connected with their families after their diagnosis even though they feared the future.
**Palestine**	Women were more likely to perform SBE if they were more educated, resided in cities, were Christian, were less religious, had a first-degree relative with breast cancer, perceived higher effectiveness and benefits of SBE, and perceived lower barriers and fatalism. Disturbance in their ability to perform their chores affected the QoL of Palestinian women with BC. General thought was that cancer is a matter of fate and test that is determined by God. In addition, their faith in God “tawakkul” relieved some of the burden of the illness. In some cities in Palestine, the evil eye was the cause of BC. Major barriers to

	screening were accessibility, language and unfriendly attitudes from the specialists and personal responsibility. Other major factors affecting screening are exposure of body parts when doing the tests, fear from and that women want their husbands to love and accept them and their bodies.
**Qatar**	Generally, women living in Qatar had good knowledge about BC but did not regularly do BSE or CBE. Married women and women with higher education and income levels were significantly more likely to be aware of and to practice BCS than women who had lower education and income levels.
**Syria**	Women felt psychological discomfort (negative emotion, body image, and depressive symptoms), physical problems (acute consequences of chemotherapy and general aspects of chemotherapy), social dysfunction (social isolation and lack of marriage opportunities), and failure in the family role (mother role and sexual relationship).
**Sudan**	Female students had moderate level of knowledge on BC, but poor performance of BSE. Practicing BSE found significantly associated with higher level of education, receiving training or counseling and history of other breast disease
**UAE**	The majority of women were aware about the existence of BC screening techniques and how to perform them but did not view themselves at risk for BC and therefore, the knowledge they acquired did not seem to influence their practice of BSE, CBE or mammogram. UAE national women scored better than non-UAE national women younger women (40–49) had better scores regarding knowledge than older women (.49) The level of education was positively associated with better knowledge.<br>Women who were diagnosed with BC focused on protecting themselves form the social stigma, facing their illness with prayers and moving on with their lives.
**Yemen**	Younger Yemeni females with BC diagnosis patients were experiencing worse QOL when compared to older patients. Women scored the highest on physical well being and lowest on functional well being.

Supplementary 1. The included studies
